# Subduing the influence of PCR inhibitors on amplifying aged, degraded, and low copy number DNA: PCR enhancer cocktail-p and rescue PCR

**DOI:** 10.1371/journal.pone.0234745

**Published:** 2020-06-16

**Authors:** Brian M. Kemp, Brittany Bingham, Ryan Frome, Marie Labonte, Erica Palmer, Ella S. Parsons, Kenneth W. Gobalet, Jeffrey Rosenthal

**Affiliations:** 1 Department of Anthropology, University of Oklahoma, Norman, Oklahoma, United States of America; 2 Laboratories of Molecular Anthropology and Microbiome Research, University of Oklahoma, Norman, Oklahoma, United States of America; 3 California State University, Bakersfield, California, United States of America; 4 Far Western Anthropological Research Group, Davis, California, United States of America; University of Helsinki, FINLAND

## Abstract

PCR inhibitors are a formidable problem to the study of aged, degraded, and/or low copy number DNA. As a result, there is a need to find alternate methods that ameliorate the efficacy of PCR. In this study, we attempted to use genetic methods to identify the species of salmonid (*Oncorhynchus* spp.) remains recovered from archaeological sites along the Feather River located in northern California, United States. In the process of doing so, we compared the efficacy of a PCR enhancer cocktail called “PEC-P” and a reagent rich PCR recipe called “rescue PCR” over standard PCR. Across all treatments (full concentration and 1:10 dilute eluates subjected to standard PCR, PEC-P, and rescue PCR) species identification was possible for 74 of 93 archaeological fish specimens (79.6%). Overall, six of the 93 samples (6.5%) consistently yielded species identification across all treatments. The species of ten specimens (10.8%) were uniquely identified from amplicons produced with either PEC-P or rescue PCR or both. Notably, the species of seven samples (7.5%) were uniquely identified with standard PCR over the alternative treatments. Considering both full concentration and 1:10 dilute eluates (N = 186), standard PCR performed as well as PEC-P (p = 0.1451) and rescue (p = 0.6753). Yet, considering results from full concentration eluates alone (N = 93), PEC-P (60.2%) outperformed both standard PCR (44.1%; p = 0.0277) and rescue PCR (40.9%; p = 0.0046). Stochasticity observed in our study cautions us against choosing a “best” performing method of those explored here and suggests their respective potentials to improve success may be sample dependent. When working with samples compromised by PCR inhibitors, it is useful to have alternative methodologies for subduing the problem. Both PEC-P and rescue PCR represent useful alternative methods for the study of aged, degraded, and/or low copy number DNA samples compromised by PCR inhibitors.

## Introduction

Numerous impurities are inadvertently co-purified with DNA and can inhibit the polymerase chain reaction (PCR) [1–3]. PCR inhibitors pose an especially formidable challenge to the analysis of aged, degraded, and/or low copy number (LCN) samples [4–8]. As a result, PCR inhibitors can lead to inaccurate quantitative PCR (qPCR) results and, if present in sufficient quantities, these impurities can lead to PCR failure/false negatives.

Means of processing samples compromised by PCR inhibitors generally fall into one of two categories. In the first category, methods have been developed for the removal of PCR inhibitors during DNA extraction and purification. For example, repeat silica extraction has been demonstrated useful in this regard [7, 9]. This method relies on repeated rounds of silica-based extraction that must remove PCR inhibitors at a rate faster than its associated loss of DNA [10]. Other methods in this category include extraction with thiopropyl sepharose 6B resin, cetyltrimethylammonium bromide (CTAB), sodium dodecyl sulphate (SDS), isopropanol precipitation, and/or polyvinylpyrrolidone (PVP) [1, 11–14].

The second category of methods includes those that subdue the influence of PCR inhibitors following DNA extraction, the simplest of which is dilution of the potential inhibited DNA eluate. Another simple strategy is to employ mutant polymerases that are more tolerant to the presence inhibitors in PCRs [15, 16].

Zhang et al. [17] developed PCR enhancer cocktails that, when used in concert with mutant polymerase (i.e., Omni Taq or Omni Klentaq), permit PCR amplification even in the presence of 25% plasma, serum, or whole blood. Different versions of these cocktails are commercially available from DNA Polymerase Technology. According to the safety data sheet, PCR enhancer cocktail 1 (PEC-1) contains D-(+)-trehalose dihydrate, L-carnitine inner salt, and Brij^®^58. This enhancer has been formulated to permit PCR amplification from heparin and citrate treated blood, and from ethylenediaminetetraacetic acid (EDTA) and heparin treated plasma. PCR enhancer cocktail 2 (PEC-2) contains D-(+)-trehalose dihydrate, L-carnitine inner salt, Brij^®^58, and heparin sodium salt. PEC-2 has been formulated to permit PCR amplification from citrate and EDTA treated blood and plasma, and from serum. Both of these enhancer cocktails are also made in alternative versions designed to better permit PCR amplification of GC-rich target molecules. According to the safety data sheet, PCR enhancer cocktail 1 GC (PEC-1 GC) and PCR enhancer cocktail 2 GC (PEC-2 GC) both contain elevated levels of L-carnitine inner salt over that found in PEC-1 and PEC-2, respectively. Lastly, PCR enhancer cocktail P (PEC-P) has been formulated for PCR amplification from plant and fecal samples. The composition of this enhancer cocktail is proprietary and no safety data sheet is made available at the DNA Polymerase Technology website (https://www.klentaq.com/). Palmer et al. [18] used PEC-P to increase their success in species identification of archaeological remains of smelt and other small fishes.

Recently, Johnson and Kemp [19] developed a method called “rescue PCR” to increase success in their study of DNA recovered from the remains of various salmon species and other fishes dating 200–10,000 years before present (YBP). The concept behind rescue PCR is simple: increase the concentration of all reagents of a PCR proportionally without changing the volume or amount of template DNA. In their study, Johnson and Kemp [19] found that a 25% increase in concentration performed best when pitted against increases of 10% and 50%. Two studies have since found rescue PCR helpful in species identification of archaeological fish remains [18, 20].

In this study, we sought to identify the species of salmonid remains recovered from archaeological sites along the Feather River located in northern California, United States. In meeting this goal we also compared our success when employing PCR enhancer cocktails and rescue PCR over standard PCR.

## Materials and methods

### Samples

Fish bones examined for this study were all recovered through archaeological excavation at a single Maidu village site (CA-BUT-4185/H), possibly the historical community of *Dairtera* or *Dietcheria* [21–23], situated on the west bank of the lower Feather River near the modern town of Gridley in the southern Sacramento Valley of California, United States. The samples came from each of four, discrete, depositional strata radiocarbon-dated to a 100 year-long period, between AD 1770 and 1870. Most of the sampled bones are vertebrae (n = 49) or indeterminate elements (n = 24), followed by post-cranial bones (n = 15), gill rakers (N = 4), and one urohyal. All were morphologically identified as Chinook salmon (*Oncorhynchus tshawytscha*) or as *Oncorhynchus* spp.

The collection will be housed at the California Department of Parks and Recreation Statewide Museums Collections Center, McClellan, California. No accession number has been assigned. The sample numbers found in S1 Table are the catalog numbers of the specimens. Note that the consulting Native American Tribe can request that the entire archaeological collection be repatriated and it may not be curated; no decision has been made at this time.

All necessary permits were obtained for the described study, which complied with all relevant regulations. Necessary permits included:

US Army Corp of Engineers 408 permits: March 19, 2014, Permit Nos 18793–2 and 18793–3.
US Army Corp of Engineers 404 permits: August 23, 2013 Permit No SPK-2012-00979, February 28, 2014 Permit No SPK-2012-00979, August 22, 2016 Permit No SPK-2015-01072.
Central Valley Flood Protection Board Encroachment, July 24, 2013, Permit No 18793–1.

Five Pacific salmon and steelhead rainbow trout (*Oncorhynchus mykiss*) have been documented from the Sacramento-San Joaquin River system of California [24]. Vertebrae are frequently the most likely recovered identifiable elements from archaeological sites [25]. Discriminating between these salmonids based on their typically fragmentary vertebrae can be notoriously challenging [26–28]. Making identifications without corroborating evidence is, thus, problematic. Prior to the evaluation of the fish bones from the Feather River archaeological sites, 49 otoliths were identified as Chinook salmon and none from other salmonids from then designated archaeological site FR-14 in Butte County provided by Far Western Anthropological Research Group, Inc. Casteel [29, 30] has demonstrated that five species of Pacific salmon can be discriminated on the basis of their otoliths. Since no salmon otoliths other than Chinook were observed, it follows that the vertebrae recovered from the same sites would be from Chinook salmon as well. This and the morphological distinctions of vertebrae described by Gobalet et al. [26] were the basis of the identifications of the salmon vertebrae as Chinook.

### DNA extraction

All pre-PCR activities were conducted in the ancient DNA (aDNA) facility at the Laboratories of Molecular Anthropology and Microbiome Research (LMAMR; lmamr.org) at the University of Oklahoma. This facility is a dedicated workspace for processing aged, degraded, and/or LCN DNA samples. Precautions aimed to minimize and monitor the introduction of contamination are practiced in the laboratory.

DNA was extracted from 93 fish remains. Approximately 50 mg or less of bone material was subsampled from each specimen with a single-use razor blade (S1 Table). If the whole bone weighed ≤ 50 mg, it was exhausted for DNA extraction. All of the samples taken for this study were submerged in 6% (w/v) sodium hypochlorite for 4 minutes [31]. The sodium hypochlorite was poured off and the samples were quickly submerged in DNA-free water twice.

The bone samples were transferred to 1.5 mL tubes, to which aliquots of 500 μL of 0.5 M EDTA were added, and the tubes gently rocked at room temperature for >48 hours. An extraction negative control, to which no bone material was added, accompanied each batch of extractions, typically in a ratio of 1:7 with the samples. This control allows us to determine if contamination was introduced during the extraction.

DNA was extracted following the method described by Kemp et al. [7]. Ninety μL of proteinase K (BIOBASIC cat # 32181) at a concentration of 1 mg/30 μL (or >20 Units/30 μL) was added to each sample, and the tubes incubated at 64–65°C for 3 hours. Following proteinase K digestion, the tubes were centrifuged at 15,000 revolutions per minute (rpm) for one minute to pellet any undigested bone, dirt, and/or “sludge”. All centrifugation steps in this study were conducted with an Eppenddorf centrifuge 5424. The liquid was carefully moved to a new 1.5 mL tube, to which 750 μL of 2.5% (w/v) “resin” (i.e., 2.5% celite in 6M guanidine HCl) and 250 μL of 6M guanidine HCl were added. The tubes were vortexed multiple times over approximately a 2 minute period.

Promega Wizard minicolumns were attached to 3 mL luer-lok syringe barrels (minus the plunger) and placed on a vacuum manifold. Three mL of DNA-free water was first pulled across the columns with the intent to wash away potential contaminating DNA. The DNA/resin mixture was subsequently pulled across the columns. The silica pellet on the minicolumns was rinsed by pulling 3 mL of 80% (v/v) isopropanol across the columns.

The minicolumns were then placed in new 1.5 mL tubes and centrifuged at 10,000 rpm for 2 minutes to remove excess isopropanol. The minicolumns were transferred to new 1.5 mL tubes. Fifty μL of DNA-free water heated to 64–65°C was added to the minicolumns and left for 3 minutes before centrifugation of the tubes for 30 seconds at 10,000 rpm. This step was repeated to yield 100 μL of extracted DNA.

### Salmonid mtDNA PCR

Full concentration DNA eluates and 1:10 dilutions of those eluates (with water) were screened for the presence of a 181 base pair (bp) fragment of the 12S gene of the mitochondrial genome that contains a 148 bp sequence informative enough to discriminate all of the Pacific salmonids (*Oncorhynchus* spp.) to the species level [15, 28, 32–34]. This system has also been demonstrated to be useful in the identification of other bony and cartilaginous fishes [e.g., 18].

Fifteen μL PCR reactions contained 1X Omni Klentaq Reaction Buffer (including a final concentration of 3.5 mM MgCl_2_), 0.32 mM dNTPs, 0.24 μM of each primer, 0.3 U of Omni Klentaq LA polymerase, and 1.5 μL of template DNA. Following denaturing at 94°C for 3 minutes, 60 cycles of PCR was conducted at 94°C for 15 s, 55°C for 15 s, and 68°C (note that this is the optimal extension temperature for Omni Klentaq LA polymerase) for 15 s. Finally, a 3 minute extension period at 68°C was conducted prior to bringing the reactions to 10°C. For the remainder of this study, we refer to these PCR reactions as “standard”. Note that the reverse primer was originally described in the incorrect orientation by Jordan et al. [32]. The corrected primers are “OST12S-F” (5’-GCTTAAAACCCAAAGGACTTG-3’) and “OST12S-R” (5’-CTACACCTCGACCTGACGTT-3’)]. Positive controls of sockeye salmon (*Oncorhynchus nerka*), added in the PCR laboratory just prior to running the reactions, accompanied all PCRs to monitor for possible failure. PCR negatives also accompanied batches of amplification to allow us to monitor for the presence of contaminating DNA.

To confirm successful amplification, 3 μL of PCR products were separated on 2% agarose gels stained with GelRed. All amplicons produced in this study were directly sequenced in both directions at Molecular Cloning Laboratories (MCLAB, South San Francisco, CA). Sequences were aligned to a rainbow trout (*O*. *mykiss*) full mitochondrial genome reference sequence [Genbank accession DQ288271; 35] in Sequencher v5.4.6. Salmonid species determinations were made following Jordan et al. [32] (see their Table 2). All non-salmonid taxa sequences were identified through National Center for Biotechnology Information’s (NCBI) BLAST function.

### Inhibition test

Full concentration DNA eluates were also tested for the presence of PCR inhibitors following the rationale of Kemp et al. [7] using a “turkey collective” as the aDNA positive control. DNA recovered from seven or more archaeological turkey (*Melleagris gallopavo*) bones [36] was pooled together to make the turkey collective. The choice to pool these individual extractions was made with the intention to reduce variation between DNA eluates in both endogenous turkey mtDNA copy number and possible inhibitors co-extracted with the turkey DNA. Before they are used in experiments, each turkey collective is demonstrated to PCR amplify six or more times in a row, hence serving as a positive control.

Fifteen μL PCRs, which included 1.5 μL of turkey collective DNA, were conducted to amplify a 186 bp portion of turkey displacement loop (D-loop) using the primers “T15709F” and “T15894R” described by Kemp et al. [36]. The components of these reactions and their cycling conditions were identical to those described above under “Salmonid mtDNA PCR”, except that annealing was conducted at 60°C and the reactions were spiked with 1.5 μL of the ancient salmon template DNA (totaling 16.5 μL reactions). The extraction negative controls were also tested for inhibitors in this manner.

These PCRs were run in parallel with reactions that contained only turkey collective DNA. These reactions served as positive controls and allowed us to preclude PCR failure from contributing to our results. PCR negatives, to which neither turkey or salmonid DNA was added, also accompanied each round of amplification. These reactions allowed us to monitor for possible contamination.

If the turkey collective failed to amplify when spiked with any given ancient salmonid DNA eluate, we considered that eluate to be inhibited. In the case that spiking the ancient salmonid DNA permitted amplification of the turkey collective DNA, we consider that DNA eluate to be inhibitor “free”.

### Repeat silica extraction of the full concentration extracts

Full concentration eluates deemed to be inhibited using the test outlined above were subjected to repeat silica extraction [7]. To the remaining volume of the eluate, 750 μL of 2.5% (w/v) resin and 250 μL of 6M guanidine HCl were added. The samples were vortexed numerous times over a 2 minute period. The extraction then followed that described above under “DNA Extraction”, except that the volume used to elute the DNA from column matched the volume being repeat silica extracted. For example, if the starting volume was 97 μL, 48.5 μL of DNA-free water heated to 65°C was added to the minicolumns and left for 3 minutes before centrifugation. This step was repeated twice for a total volume of 97 μL.

These repeat silica eluates were tested again for the presence of salmonid mtDNA and inhibition as described above. Those still deemed to be inhibited were once again repeat silica extracted, and tested for salmonid mtDNA and inhibition. This was carried out until all full concentration eluates: (1) either produced a positive result in the salmonid mtDNA reaction, or (2) were deemed to be free of inhibition but failed to amplify in the salmonid mtDNA reaction. In the latter case, the samples were concluded to not contain amplifiable salmonid mtDNA using standard PCR.

### PCR enhancer cocktails

While the PCR enhancer cocktail datasheet calls for reactions at 50% (v/v) (klentaq.com), personal communication with Wayne Barnes (of DNA Polymerase Technology) led us to initially test PEC-1, PEC-2, and PEC-P in PCRs at 20% (v/v) on subsets of samples. Fifteen μL PCR reactions contained 1X Omni Klentaq Reaction Buffer (including a final concentration of 3.5 mM MgCl_2_), 0.32 mM dNTPs, 0.24 μM of each primer, 0.3 U of Omni Klentaq LA polymerase, 20% (v/v) PCR enhancer cocktail, and 1.5 μL of template DNA.

We were satisfied enough with the performance of PEC-P over PEC-1 and PEC-2 (see Results) to test this PCR enhancer cocktail on all of the full concentration eluates that had been carried through the inhibition test as described above. PEC-P was also tested on the 1:10 dilutions of the original eluates.

DNA sequencing, alignment, and identification followed that described under “Salmonid mtDNA PCR” above.

### Rescue PCR

Rescue PCR at a 25% increase was carried out as described by Johnson and Kemp [19] on all of the full concentration eluates that had been carried through the inhibition test as described above, as well as on the 1:10 dilution of the original eluates. Fifteen μL rescue PCR reactions contained 1.25X Omni Klentaq Reaction Buffer (including a final concentration of 4.375 mM MgCl_2_), 0.4 mM dNTPs, 0.3 μM of each primer, 0.375 U of Omni Klentaq LA polymerase, and 1.5 μL of template DNA. DNA sequencing, alignment, and identification followed that described under “Salmonid mtDNA PCR” above.

### Statistical analysis

Chi square tests were used to compare success rates in species identification using standard PCR, PEC-P, and rescue PCR. An alpha level of 0.05 was set as the cut-off for statistical significance of the tests.

## Results

For our initial experiments with the three PCR enhancer cocktails, PEC-1 treatment yielded species identification on five of 14 eluates (1124–1, 1657–3, 1657–5, 1124–1 1:10, and 1657–5 1:10) (Table 1). Standard PCR yielded identification on six of 14 of the same eluates (1657–2, 1657–3, 1657–5, 1124–1 1:10, 1124–2 1:10, and 1657–5 1:10). It is notable that PEC-1 uniquely identified the 1124–1 full concentration sample as Chinook salmon and standard PCR uniquely identified the 1124–2 1:10 sample as Chinook salmon.

**Table 1:** Results of initial experiments with PEC-1, PEC-2, and PEC-P.

Sample	Concentration	Number of Repeat Silica Extractions	Standard	PEC-1
1124–1	full	0		Chinook
1124–2	full	0		
1657–1	full	1		
1657–2	full	2	Chinook	
1657–3	full	0	Chinook	Chinook*
1657–4	full	1		
1657–5	full	0	Sucker	Sucker
1124–1	1:10	n/a	Chinook	Chinook
1124–2	1:10	n/a	Chinook	
1657–1	1:10	n/a		
1657–2	1:10	n/a		
1657–3	1:10	n/a		
1657–4	1:10	n/a		
1657–5	1:10	n/a	Sucker	Sucker
			**Standard**	**PEC-2**
1002–1	full	0		
1611–1	full	1	Chinook	
1611–2	full	0		
1611–3	full	0		
1611–4	full	0		Sucker
1611–5	full	0		
1724–1	full	0	Chinook	
1002–1	1:10	n/a		Chinook
1611–1	1:10	n/a		
1611–2	1:10	n/a		
1611–3	1:10	n/a		Chinook
1611–4	1:10	n/a	Sucker	
1611–5	1:10	n/a		
1724–1	1:10	n/a		
			**Standard**	**PEC-P**
1700–1	full	0		Chinook*
1700–2	full	0		Chinook
1700–3	full	0		Chinook
1700–4	full	0		Chinook
1700–5	full	0	Chinook	Chinook
1700–6	full	0	Chinook	
1700–7	full	0		
1700–1	1:10	n/a	Chinook	
1700–2	1:10	n/a		Chinook
1700–3	1:10	n/a	Chinook	Chinook
1700–4	1:10	n/a	Chinook	
1700–5	1:10	n/a		Chinook*
1700–6	1:10	n/a		
1700–7	1:10	n/a	Chinook	

Here results of species identification are reported for samples at full concentration (noting the number of repeat silica extractions required for a sample to be uninhibited according to our inhibition test) and at 1:10 dilution. Blank cells indicate no DNA amplification and, thus no species identification. We were satisfied with the results obtained with PEC-P to further test this PCR enhancer cocktail on the full set of samples included in this study.

*Denotes samples for which the reverse sequence failed, but where species identification was still possible.

PEC-2 treatment yielded species identification on three of 14 eluates (1611–4, 1002–1 1:10, and 1611–3 1:10) (Table 1). These are three eluates that failed to yield species identification using standard PCR. In this experiment, standard PCR was used to identify the species of three of 14 eluates, notably three eluates that were not aided by the addition of PEC-2 (1611–1, 1724–1, and 1611–4 1:10).

PEC-P treatment yielded species identification on eight of 14 eluates, five of which were uniquely identified using this enhancer (1700–1, 1700–2, 1700–3, 1700–4, and 1700–5 1:10) (Table 1). Of these same 14 eluates, standard PCR was used to identify the species of six of them, three of which were uniquely identified with standard PCR (1700–6, 1700–1 1:10, and 1700–7 1:10).

While additional experiments using PEC-1 and PEC-2 are clearly warranted, we focused our efforts on further exploring the utility of PEC-P in improving our ability to determine the species of these archaeological fish remains.

Of the 93 full concentration eluates, 33 required at least one repeat silica extraction to be deemed uninhibited (range 1–3; average 1.8) (S1 Table). Across all of the treatments (full and 1:10 dilute eluates subjected to standard PCR, PEC-P, and rescue PCR), species identification was possible for 74 of 93 samples (79.6%). Of the 74 identifications, 69 were determined to be Chinook salmon (93.2%). One Chinook sample (219–1) exhibited a lineage lacking the C>T transition at np 711 that is common to Chinook salmon. As this sequence motif was repeated three times, this difference is not likely to be the product of post-mortem damage [37, 38]. As we found no complete match to this lineage in Genbank, it is possible that this lineage has yet to be sampled from extant populations or it has gone extinct. One sample (1277–3) was identified as rainbow trout or steelhead (*O*. *mykiss*) and another sample (1572–2) as sockeye salmon (*O*. *nerka*).

The remaining three samples were identified as non-salmonid species, the sequences from which were subjected to a basic local alignment search tool (BLAST) of Genbank. The 12S sequences of these specimens most closely resemble examples of suckers (*Catostomus spp*.), and are presumably Sacramento suckers (*Catostomus occidentalis*) given their origination on the Feather River. However, comparative 12S sequence data are unavailable for Sacramento suckers to evaluate this possibility. Aligned to a comparative full mitochondrial genome of a longnose sucker (*Catostomus catostomus*, Genbank accession number NC037013), samples 1611–4 and 1657–5 exhibit G>A transitions at np 650 and sample 1626–2 exhibits G>A transitions at nps 650 and 709.

As detailed in S1 Table, possible post-mortem sequence damage was observed in a few of the sequences. Sample 597–6 (amplified 1:10 with rescue PCR) revealed a C>T “transition” at np 610 and sample 1611–1 (amplified at full concentration with standard PCR) revealed a C>T “transition” at np 626 over the expected mutational motif for a Chinook salmon [32]. As no addition amplifications were possible from these two samples, we cannot rule out the possibility that these “mutations” are artifacts of post-mortem damage [37, 38]. Sample 1501–2 (amplified 1:10 with standard PCR) exhibited a G>A “transition” at np 687 and sample 1700–6 (amplified 1:10 with rescue PCR) exhibited C>T “transitions” at nps 640 and 699. These “mutations” were not observed from additional amplifications of these specimens, indicating that they are likely the product of post-mortem degradation. Despite these observations of damage or possible damage, species identification was still possible for these specimens and, thus, we treated them as successes in comparing the outcomes of the experimental treatments.

A 1:10 diluted extraction negative control tested positive under standard PCR conditions, the sequence of which matched Chinook salmon. This extraction negative control is associated with seven samples (219–1, 219–2, 219–3, 219–4, 597–1, 597–2, and 1310–3). Where the 1:10 diluted extraction negative control tested positive, three of the 1:10 diluted eluates (samples 219–2, 219–4, and 1310–3) were also identified as Chinook salmon. Neither of the two PCR negatives associated with this PCR amplified. This extraction negative control failed to amplify in five other attempts: 1) at full concentration with standard PCR, 2) at full concentration and at 1:10 dilution with PEC-P, and 3) at full concentration and at 1:10 dilution with rescue PCR. In all, we do not believe these observations indicate that this batch of extractions is contaminated. It is more likely that our observation of contamination from the 1:10 diluted extraction negative control amplified under standard PCR conditions was the product of cross contamination while setting up this particular set of reactions. It is also notable that samples 219–2, 219–4, and 1310–3 that tested positive alongside this extraction negative control where identified as Chinook under different amplification conditions. Nevertheless, as no other positive results were observed across the many extraction and PCR negative controls in our study, contamination was not pervasive.

The assumptions concerning the morphological identification of the remains as Chinook salmon are not perfect as is illustrated by the molecular identification of sockeye salmon (sample 1572–2) and rainbow trout (sample 1277–3) that were morphologically identified as Chinook salmon. Following the molecular analyses, co-author Rosenthall provided co-author Gobalet with images of the elements identified as sucker by molecular evaluation (samples 1611–4, 1626–2, and 1657–5). Based on the fragmentary nature of these bones, they cannot be morphologically identified to a fish taxon. Gobalet did not recall having looked at the bones and, thus, cannot explain how they were designated initially as Chinook salmon.

Nevertheless, the species profile of these specimens is consistent with their origination along the Feather River. Historically, the Feather River supported one of the largest populations of Chinook salmon in the Central Valley of California divided between fall and spring spawning runs [39]. Likewise, of the 32,000 morphologically identified fish bones from nine archaeological sites sampled along the river, 43% were identified as salmonid and 40% as sucker [i.e., Sacramento sucker (16%) or carp/minnow/sucker (Cypriniformes) (24%)].

Across all treatments (full concentration and 1:10 eluates subjected to standard PCR, PEC-P, and rescue PCR), six of the 93 samples (6.5%) consistently yielded species identification (132–1, 219–3, 913–1, 1644–3, 1700–9, 1738–1) (S1 Table). The species of seven samples were uniquely identified with standard PCR on full concentration eluates over the alternative treatments (samples 44–1, 1124–3, 1277–1, 1572–2, 1611–1, 1626–5, and 1657–2). The species of ten specimens were uniquely identified with either PEC-P (samples 597–4 and 1611–5) or rescue PCR (samples 253–2 and 597–6) or both (samples 1002–1, 1072–1, 1215–1, 1501–3, 1611–3, and 1700–2) over standard PCR.

Standard PCR permitted species identification from 41 of the 93 full concentration eluates (44.1%) and 37 of 93 of the 1:10 dilutions (39.8%) (Table 2 and S1 Table). Thus, identification with standard PCR was made possible from 78 of 186 eluates (41.9%). Treatment with PEC-P permitted species identification from 56 of the 93 full concentration eluates (60.2%) and 36 of 93 of the 1:10 dilutions (38.7%). Overall identification with PEC-P was made possible from 92 of 186 eluates (49.5%). Treatment with rescue PCR yielded species identification of 38 of the 93 full concentration eluates (40.9%) and 44 of 93 of the 1:10 dilutions (47.3%). Overall, rescue PCR made possible species identification from 82 of 186 eluates (44.1%).

**Table 2:** Success rates in species identification achieved with three amplification approaches on full concentration and 1:10 diluted eluates.

	Full Concentration	1:10 Dilution	Overall
Standard PCR	41/93 (44.1%)	37/93 (39.8%)	78/186 (41.9%)
PEC-P	56/93 (60.2%)	36/93 (38.7%)	92/186 (49.5%)
Rescue PCR	38/93 (40.9%)	44/93 (47.3%)	82/186 (44.1%)

See S1 Table for more details.

A chi square test indicates that standard PCR performed just as well as rescue PCR at full concentration (44.1% vs. 40.9%; p = 0.6563), 1:10 (39.8% vs. 47.3%; p = 0.3006), and overall (41.9% vs. 44.1%; p = 0.6753). PEC-P treatment performed better (60.2%) than standard PCR (44.1%) on full concentration eluates (p = 0.0277), but equally well on 1:10 eluates (38.7% vs 39.8%; p = 0.8806) and overall, as well (49.5% vs 41.9%; p = 0.1451). PEC-P treatment also performed better (60.2%) than rescue PCR (40.9%) on full concentration eluates (p = 0.0046), but equally well on 1:10 eluates (38.7% vs. 47.3%) p = 0.2361) and overall (49.5% vs. 44.1%) p = 0.2988).

## Discussion

The inadvertent co-purification of PCR inhibitors with DNA presents an especially formidable problem to the study of aged, degraded, and/or LCN DNA. In addition to yielding inaccurate quantitative PCR (qPCR) results, if present in sufficient quantities, these impurities can lead to PCR failure/false negatives.

In comparison to “standard” PCR, in this study we sought to evaluate the efficacy of the addition of a PCR enhancer cocktail [PEC-P; see klentaq.com and 17] and a reagent rich PCR recipe called rescue PCR [19] in the pursuit of species identification of various archaeological fish remains. Overall, for full concentration eluates and 1:10 dilutions, PEC-P and rescue PCR yielded equivalent results to that of standard PCR. However, considering results from the full concentration eluates alone, PEC-P outperformed both standard and rescue PCR. Because the chemical composition of PEC-P is proprietary, we cannot speculate on the mechanism by which this enhancer works. But, it is notable that PEC-P was designed to work well with plant and fecal samples. It must be that our archaeological samples contain similar inhibitors or classes of inhibitors to feces and plants.

It is unclear why rescue PCR did not perform especially well, as two previous studies benefited greatly from inclusion of this method [18, 20]. It is likely the product of us working with DNA eluates that contain unknown amounts of unknown inhibitors. Future controlled studies of inhibition will be useful in determining under what conditions rescue PCR works most well.

It is notable, however, that these alternative approaches produced ten unique identifications in our study over that for standard PCR. Conversely, standard PCR produced seven unique species identifications over PEC-P and rescue PCR. Given this pattern and the fact that, in some cases, PCRs from diluted eluates outperformed those from full concentration eluates or vice versa (Table 1 and S1 Table), suggests that choosing a “best” performing method of those explored here may be sample dependent.

Thus, when working with samples compromised by PCR inhibitors, it is useful to have alternative methodologies for subduing the problem. PEC-P and rescue PCR represent only two of such methods. We have incorporated both of them into our standard workflow for ancient DNA and continue to find them useful. In general we recommend that researchers pay close attention to the role that PCR inhibitors may play in their studies.

## Supporting information

S1 Table Samples and results of species identification using standard PCR, PEC-P, and rescue PCR. Blank cells indicate no DNA amplification and, thus no species identification. Asterisks (*) denote samples for which the reverse sequence failed, but where species identification was still possible. Dagger (†) denotes samples for which the forward sequence failed, but where species identification was still possible. Double dagger (‡) denotes a sequence with a gap in the center, but where species identification was still possible. Section sign (§) indicates the observation of possible post-mortem damage in the sequence at the position(s) noted relative to a rainbow trout mitochondrial genome reference sequence [Genbank accession DQ288271; 35]. To aid in interpreting this table a few examples are worth detailing. For example, weighing 51.3 mg, sample 44–1 required two rounds of repeat silica extraction to sufficiently remove inhibition and was identified as Chinook salmon with standard PCR, but failed to amplify with any other treatment (dilution, PEC-P, or rescue PCR). In a second example, the full concentration eluate of sample 597–6 was deemed to be uninhibited and was uniquely identified as Chinook salmon when the 1:10 eluate was amplified with rescue PCR. This amplicon revealed possible damage with a C>T “transition” observed at np 610. In the last example, the full concentration eluate of sample 1501–2 was deemed to be uninhibited. Species identification of this sample was possible using standard PCR on the 1:10 dilute eluate and at full concentration using PEC-P and rescue PCR. The amplicon produced from the 1:10 dilute eluate revealed G>A damage at np 687.
(XLSX)
